# Growth Hormone Reverses Cisplatin‐Induced Ovarian Reserve Depletion in Mice: Transcriptomic Elucidation of Mitochondrial Metabolic Reprogramming and Protein Synthesis Regulation

**DOI:** 10.1096/fba.2026-00193

**Published:** 2026-09-23

**Authors:** Tianhong Huang, Niu Wang, Dongsheng Xiong, Yanan Zhang, Jiajing Wei, Furui Chen, Yu Qiu, Fangyi Long, Qianwen Luo, Jesse Li‐Ling, Yan Gong

**Affiliations:** ^1^ Department of Medical Genetics and Prenatal Diagnosis Sichuan Provincial Women's and Children's Hospital, the Affiliated Women's and Children's Hospital of Chengdu Medical College Chengdu Sichuan China; ^2^ Center of Reproductive Medicine Sichuan Provincial Women's and Children's Hospital, the Affiliated Women's and Children's Hospital of Chengdu Medical College Chengdu Sichuan China; ^3^ Department of Obstetrics Sichuan Jinxin Xinan Women's and Children's Hospital Chengdu Sichuan China; ^4^ Center of Laboratory Medicine Sichuan Provincial Women's and Children's Hospital, the Affiliated Women's and Children's Hospital of Chengdu Medical College Chengdu Sichuan China; ^5^ Department of Medical Genetics West China Second University Hospital, Sichuan University Chengdu Sichuan China

**Keywords:** decreased ovarian reserve, growth hormone, mitochondrial metabolic reprogramming

## Abstract

Diminished ovarian reserve (DOR) presents a formidable challenge for assisted reproduction technology, as affected patients typically exhibit reduced oocytes and embryos during in vitro fertilization and embryo transfer (IVF‐ET), which may ultimately lead to compromised clinical pregnancy rate. Although growth hormone (GH) supplementation has shown clinical efficacy in improving the outcomes of IVF‐ET, the precise molecular mechanisms underlying its ability to enhance ovarian reserve have remained poorly characterized. In this study, a cisplatin‐induced DOR mice model was established via intraperitoneal cisplatin administration. GH was subsequently evaluated at four dose levels over a 21‐day intervention period. Comprehensive assessments included quantitative follicular histomorphometry, serum hormone profiling, and whole‐transcriptome analysis of ovarian tissue. GH treatment significantly increased the number of primary and secondary follicles in a dose‐dependent manner (*p* < 0.05), while concurrently reducing atretic follicle counts in the medium‐ and high‐dose GH groups compared to the DOR group (*p* < 0.05). Furthermore, GH treatment restored hormonal balance, characterized by decreased follicle‐stimulating hormone (FSH) and increased estradiol (E_2_) and anti‐müllerian hormone (AMH) levels (*p* < 0.05). Transcriptomic analysis revealed that differentially expressed genes (DEGs) between the DOR and negative control (NC) groups were primarily associated with mitochondrial energy metabolism and the reproductive endocrine system, with functional enrichment in ribosome biogenesis, oxidative phosphorylation, and thermogenesis. In contrast, DEGs between the high‐dose GH and DOR groups were predominantly enriched in pathways related to immunity, mitochondrial function, protein synthesis, and extracellular matrix‐receptor interactions. In conclusion, GH can restore the ovarian reserve in cisplatin‐induced DOR mice in a dose‐dependent manner, potentially by modulating the genetic networks involved in energy metabolism, oxidative homeostasis, and protein synthesis.

## Graphical abstract

This study investigated the effects of different doses of growth hormone (GH) on cisplatin (CDDP)‐induced ovarian injury and the underlying mechanisms in female Kunming mice. First, a diminished ovarian reserve (DOR) model was established by intraperitoneal injection of 1.5 mg/kg CDDP for 7 consecutive days. A 21‐day treatment phase followed: the control group received 0.2 mL saline, while the low‐, middle‐, and high‐dose GH groups received 0.4, 0.8, and 1.6 mg/kg GH, respectively. At the end of treatment (Day 28), samples were collected after 12 h of fasting: orbital blood for measurement of AMH, FSH, and estradiol (E_2_); mice were then euthanized to harvest ovarian tissue. Histology was performed with HE staining to evaluate ovarian cortex and medulla thickness and follicle counts (*n* = 6 per group). For transcriptomics, RNA‐seq was conducted on ovarian tissue (*n* = 3 per group) to identify differentially expressed genes (DEGs) and analyze enriched biological processes and signaling pathways. The main findings indicate that GH mitigates CDDP‐induced ovarian damage in a dose‐dependent manner, preserving tissue architecture, normalizing hormone levels, and promoting beneficial remodeling of the transcriptome.

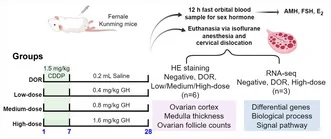

**Abbreviations**
AMH: anti‐müllerian hormone
BP: biological process
CC: cellular component
CDDP: cisplatin
CTX: cyclophosphamide
DEGs: differentially expressed genes
DOR: diminished ovarian reserve
E_2_
: estradiol
FSH: follicle‐stimulating hormone
GH: growth hormone
GO: gene ontology
IVF‐ET: in vitro fertilization and embryo transfer
KEGG: kyoto encyclopedia of genes and genomes
MF: molecular function
NC: negative control
OS: ovarian stimulation
POF: premature ovarian failure

## 1 Introduction

Patients with diminished ovarian reserve (DOR) undergoing in vitro fertilization and embryo transfer (IVF‐ET) frequently exhibit poor ovarian response, characterized by reduced oocyte yield, fewer viable embryos, and lower clinical pregnancy rate [1, 2]. Various therapeutic interventions have been explored to improve the outcomes in this population, including pharmacological pretreatment (such as growth hormone [GH], coenzyme Q10, dehydroepiandrosterone, and traditional Chinese medicine), optimization of ovarian stimulation (OS) protocols, and luteinizing hormone supplementation during OS. However, the clinical efficacy of these strategies remains controversial [1, 3]. Among these interventions, GH has been extensively investigated, with several large‐scale studies evaluating its potential benefits [4, 5].

GH is a 191‐amino acid, single‐chain polypeptide primarily secreted by the anterior pituitary gland [4, 6, 7]. In females, GH secretion declines gradually after puberty and more markedly after the age of 30 [6, 8]. The coadministration of GH and gonadotropin during OS was first reported in 1988 [9]. Subsequent studies have suggested that GH supplementation may enhance ovarian response, improve the quality of oocytes and embryos, and increase endometrial receptivity. However, its impact on live birth rate remains controversial [4, 10, 11, 12]. Notably, while GH treatment may improve rates of clinical pregnancy and live birth in patients aged 35–39, similar benefits have not been consistently observed in women over 40 [13]. Due to ethical constraints in human research, direct evidence and mechanistic insights into the role of GH in restoring ovarian reserve are limited, necessitating the use of well‐validated DOR animal models.

Murine models are the predominant experimental systems for studying DOR, utilizing approaches such as natural aging, chemical induction (cyclophosphamide [CTX] or cisplatin [CDDP]), metabolic disruptions, radiation, genetic modification, and exposure to environmental toxicants [14, 15, 16]. Although GH has been shown to enhance folliculogenesis and oocyte maturation while reducing apoptosis in naturally aging models, this model has significant drawbacks: (1) the prolonged timeframe required (typically > 32 weeks), (2) low experimental feasibility for large‐scale studies, and (3) a limited therapeutic response to GH, mimicking the poor outcomes seen in women over 40 [4, 17, 18]. Conversely, chemotherapy‐induced models in young mice (6–8 weeks old) offer rapid induction (1–5 weeks), high reproducibility, cost‐effectiveness, and operational convenience [14, 19, 20]. Previous studies have shown that medium (0.8 mg/kg/d) to high (1.6 mg/kg/day) doses of GH can significantly enhance follicular development in CTX‐induced premature ovarian failure (POF) models [21], while Kim et al. [20] reported that high‐dose GH treatment (2.0 mg/kg/day) was required to effectively preserve the primordial follicle pool and improve folliculogenesis. Therefore, the optimal therapeutic dose of GH and the precise molecular mechanisms remain to be fully elucidated.

GH impact on folliculogenesis and oocyte quality via both direct and indirect pathways has remained to be fully elucidated [4, 20, 21]. By binding to the GH receptor, GH can directly improve mitochondrial function and oocyte maturation [22]. Indirectly, GH can interact with GH receptors on granulosa cells to increase sensitivity to gonadotropin, promote steroidogenesis, stimulate proliferation and differentiation, and inhibit the apoptosis of granulosa cells [23, 24]. Although pathways such as the JAK–STAT, Notch (Notch1, CBF1, HES1), and PI3K/Akt have been implicated [21, 25, 26], comprehensive molecular characterization is still lacking. By building upon these findings, this study was designed to: (1) systematically evaluate the dose‐dependent effects of GH on ovarian reserve in CDDP‐induced DOR models, (2) elucidate the molecular mechanisms through RNA‐seq analysis, and (3) ultimately translate these findings to optimize the outcomes of IVF‐ET in DOR patients.

## 2 Materials and Methods

### 2.1 Experimental Animals

Animal experiments were carried out in accordance with the ARRIVE guidelines. This study has been approved by the Animal Ethics Committee of Chengdu Medical College (approval no. 2023008). All methods used in this study were carried out in accordance with relevant guidelines and regulations.

Young (6–8 week‐old) female Kunming mice (Chengdu Dashuo Experimental Animals Co. Ltd.) were group‐housed in the SPF laboratory animal facility of Chengdu Medical College. The mice were fed with standard SPF‐grade mice chow and purified water ad libitum. The room temperature was maintained at 22°C–26°C with a relative humidity ranging from 45% to 70%. A 12 h light/12 h dark cycle was artificially controlled (light: 7:15–19:15; dark: 19:15–7:15 the next day), and constant good ventilation was guaranteed. All mice were acclimatized for 2 weeks, and their activity, behavior, fur condition, skin laxity, and food intake were monitored daily. Thereafter, vaginal smears were obtained daily, and 60 mice in the same estrous cycle were selected for the study.

To establish the DOR model, 48 mice were intraperitoneally injected daily with 1.5 mg/kg CDDP (Jiangsu Haosen Pharmaceutical Co. Ltd.) for seven consecutive days [27]. By using computer‐generated random number table. The DOR mice were randomly assigned into four subgroups based on the GH dosage (12 each): DOR control (0 mg/kg), low‐dose (0.4 mg/kg), medium‐dose (0.8 mg/kg), and high‐dose (1.6 mg/kg) [17]. The recombinant GH (Anhui Anke Biotechnology Co. Ltd., Anhui, China) was diluted in 0.2 mL of physiological saline and injected for 21 days [21, 28].

**FIGURE 1:**
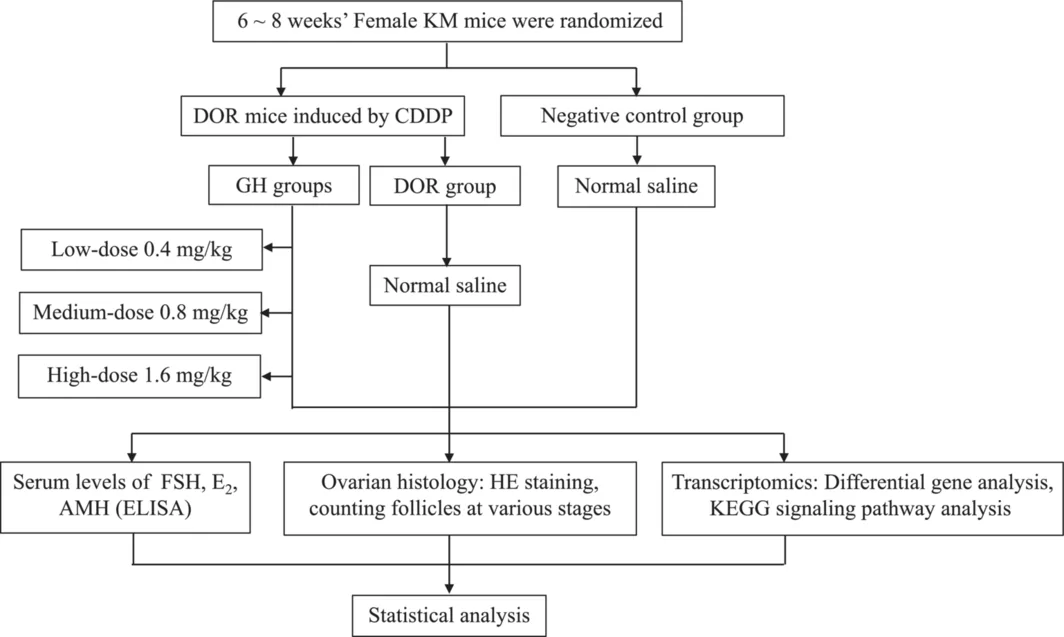
Flow diagram of the experiments.

The other 12 mice from the negative control (NC) group were intraperitoneally injected with an equal volume of physiological saline daily during DOR model establishment and GH intervention (Figure 1).

### 2.2 Measurement of Hormones in Serum

The mice were euthanized by cervical dislocation under anesthesia induced by an overdose of isoflurane, 1 day after the completion of drug administration. Blood samples were collected immediately with the retro‐orbital bleeding method and placed at room temperature for 30 min, and then centrifuged at 3000 rpm for 15 min at 4°C. The supernatant (serum) was collected and stored at −80°C. Serum levels of anti‐müllerian hormone (AMH), follicle‐stimulating hormone (FSH), and estradiol (E_2_) were measured with enzyme‐linked immunosorbent assay kits (Shanghai Zhuocai Biotechnology Co. Ltd., Shanghai, China) by following the manufacturer's protocol. Sensitivities of the three hormones were 10 pg/mL, 0.1 mIU/mL, and 0.1 pmol/L, and the reference ranges were 125–4000 pg/mL, 2.5–80 mIU/mL, and 2–64 pmol/L, respectively. The interplate coefficients of variation were all < 10%.

### 2.3 Histopathological Examination of Ovarian Tissue

Immediately after blood collection, the ovaries were dissected under sterile conditions to remove the surrounding mesentery and fat tissue. One of the ovaries from each mouse was fixed in 4% paraformaldehyde solution (Biosharp, USA) and stored at 4°C for histopathological examination, whereas the other ovary was stored in liquid nitrogen for transcription. Fixed ovaries were dehydrated through a graded series of alcohols, embedded in paraffin, and sectioned into 5 μm thick tissue slices using a microtome (Leica, Germany). After being incubated overnight, the sections were dewaxed, hydrated, and stained with Hematoxylin and Eosin. Thereafter, under a microscope (Nikon, Japan), the number of follicles at various stages was counted. The central (mid–sagittal) section was selected to measure the thickness of the ovarian cortex and medulla.

### 2.4 Transcriptomics of the Ovarian Tissue

The ovaries from the GH subgroup with the greatest improvement in hormone and ovarian morphology (High_A, High_B, High_C), DOR (DOR_A, DOR_B, DOR_C) and the NC groups (Wt_A, Wt_B, Wt_C) were selected for RNA‐Seq. Three ovarian samples from each group were used for serial manipulation of RNA, including extraction (Ambion, USA), assessment of quantity and integrity, detection of total RNA, library construction and quality inspection (Beckman Coulter, USA), sequencing (Illumina NovaSeq, PE150), data processing and analysis. The analysis has involved quality control, sequence alignment to the reference genome, quantification of gene expression, differential expression analysis, and enrichment analysis of differentially expressed genes. Gene expression levels were quantified with Fragments Per Kilobase of transcript per Million mapped fragments (FPKM) software, with Pearson correlation coefficients (*R* ^2^) exceeding 0.8 for all samples, which indicated the reliability of experimental data suitable for subsequent differential analysis. Gene Ontology (GO) enrichment analysis revealed significantly enriched functions of the differentially expressed genes, which were corrected for gene length bias. Kyoto Encyclopedia of Genes and Genomes (KEGG) pathway was analyzed with computer software (Illumina, NEB, USA) to identify the differentially expressed genes and their overall expression to elucidate the metabolic and signaling pathways involved.

### 2.5 Statistical Analysis

All data was analyzed with the SPSS 26.0 software. Quantitative data with normal distribution are expressed as means ± standard deviations (M ± SDs) and analyzed with independent‐samples *t*‐test. Transcriptomic data was analyzed with DESeq2 software to identify differential gene expression between particular groups. *p* value was adjusted with the Benjamini‐Hochberg method to derive the adjusted *p* values (*p*_adj_), where *p* < 0.05 was considered as statistically significant.

## 3 Results

### 3.1 High‐Dose GH Increased the Body Weight in DOR Mice

**FIGURE 2:**
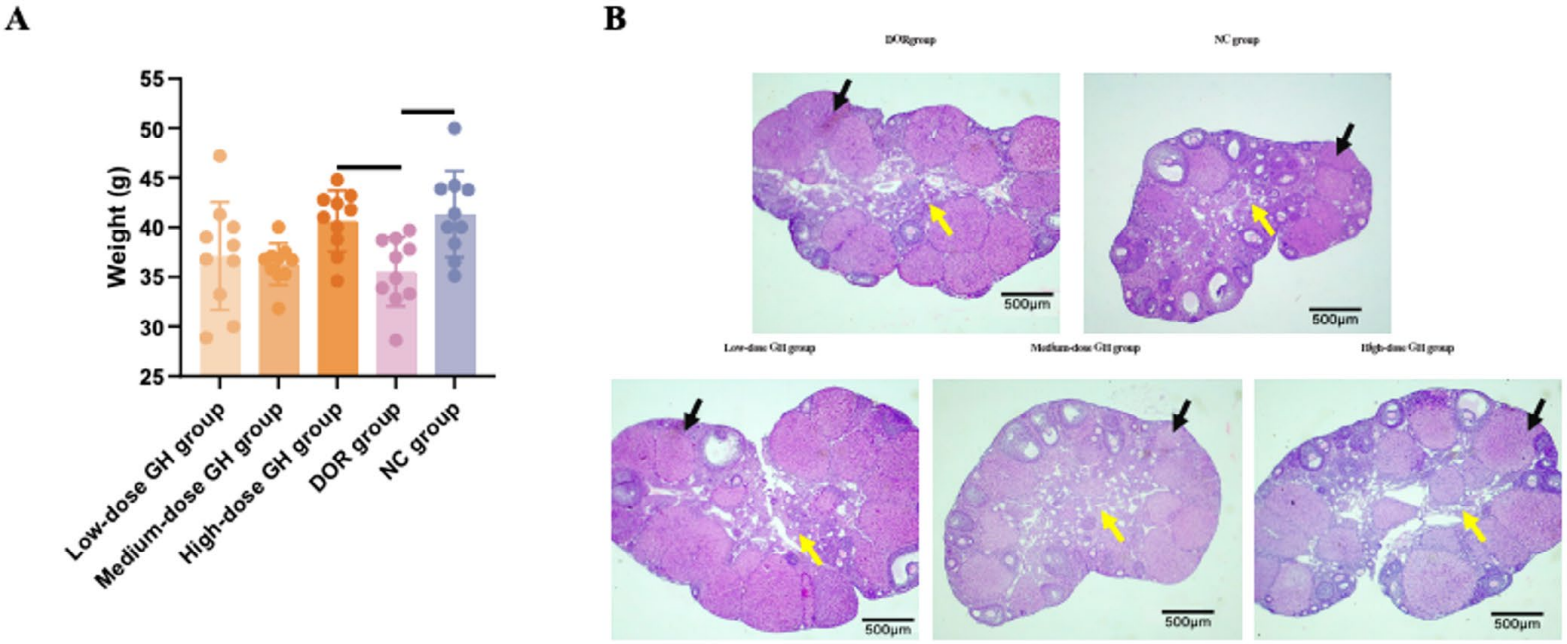
Body weight and HE staining of ovaries. (A) Comparison of weight among the groups. *Compared with the NC group, *p* < 0.05; ^#^Compared with the high‐dose GH group, *p* < 0.05. (B) Overall view of the ovaries in each group. The black arrows indicate the ovarian cortex, and the yellow arrows indicate the medullary region. The magnification is 4 × 10, and the scale bar is shown in the figure.

The mice from the CDDP‐induced DOR group exhibited a significant reduction in body weight. GH intervention mitigated this weight loss, though a significant restoration was observed only in the high‐dose GH group (*p* < 0.05) (Figure 2A).

### 3.2 High‐Dose GH Improved Serum Hormone Profiles

Compared with the NC group, the DOR group showed significantly lower serum AMH and E_2_ levels, along with markedly higher FSH levels (*p* < 0.05). Compared with the DOR group, FSH level decreased while E_2_ level increased across all GH groups. Notably, AMH level was significantly elevated only in the high‐dose GH group (*p* < 0.05). Within the GH groups, there was a trend toward increasing levels of AMH and E_2_, and decreasing levels of FSH with increasing GH doses, albeit the differences were not significant (*p* > 0.05) (Table 1).

**TABLE 1:** Comparison of serum sex hormone and AMH levels of mice from different groups.

Groups	AMH (pg/mL)	FSH (mIU/mL)	E_2_ (pmol/L)
Low‐dose GH group	2379.01 ± 283.78	46.46 ± 4.54*	35.69 ± 4.02*^,△^
Medium‐dose GH group	2397.84 ± 193.43	46.06 ± 5.74*	35.97 ± 3.44*^,△^
High‐dose GH group	2546.87 ± 138.83*	42.17 ± 6.04*	38.36 ± 2.11*^,△^
DOR group	2242.08 ± 108.30^△^	52.75 ± 4.85^△^	32.02 ± 3.81^△^
NC group	2507.51 ± 126.36	42.50 ± 6.17	42.71 ± 5.57

*Note:* Continuous variables are presented as mean ± SD. *p* < 0.05 was considered statistically significant.

*p < 0.05 compared to DOR group. ^△^ *p* < 0.05 compared to untreated group.

### 3.3 Medium‐To High‐Dose GH Improved Ovarian Morphology

Compared to the DOR group, GH‐treated mice exhibited a dose‐dependent increase in ovarian volume and improved vascularization, along with a marked reduction in inflammatory cell infiltration (Figure 2B).

Histomorphometric analysis revealed that the DOR group had significantly decreased cortical thickness and cortex/medulla ratio, but increased medullary thickness compared to the DOR group (*p* < 0.05). Conversely, high‐dose GH treatment significantly reversed these changes, resulting in increased cortical thickness and cortex/medulla ratio, and decreased medullary thickness compared to the DOR group (*p* < 0.05). Furthermore, within the GH groups, the high‐dose group showed a significantly thicker cortex and higher cortex/medulla ratio compared to the low‐dose group, and a higher cortex/medulla ratio compared to the medium‐dose group (*p* < 0.05) (Table 2).

**TABLE 2:** Comparison of ovarian cortex and medulla thickness of mice in each group.

Groups	Cortical thickness (μm)	Medullary thickness (μm)	Cortex/Medulla ratio
Low‐dose GH group	631.10 ± 129.86^#,△^	623.13 ± 133.64	1.03 ± 0.20^#,△^
Medium‐dose GH group	736.20 ± 119.00	650.47 ± 175.45^△^	1.22 ± 0.45^#,△^
High‐dose GH group	831.13 ± 244.67*	546.26 ± 203.07*	1.61 ± 0.42*
DOR group	672.70 ± 170.12^△^	733.87 ± 179.66^△^	0.92 ± 0.13^△^
NC group	858.38 ± 171.00	506.87 ± 63.35	1.73 ± 0.51

*Note:* Continuous variables are presented as mean ± SD. *p* < 0.05 was considered statistically significant.

^#^ *p* < 0.05, compared to high‐dose GH group. *p < 0.05, compared to DOR group. ^△^ *p* < 0.05, compared to NC group.

### 3.4 Medium‐To High‐Dose GH Improved Folliculogenesis

Compared with the NC group, the DOR group exhibited significantly fewer primary, secondary, and antral follicles, alongside a marked increase in atretic follicles (*p* < 0.05). The numbers of primary and secondary follicles were significantly higher across three GH groups compared to the DOR group (*p* < 0.05). Furthermore, the number of atretic follicles was significantly reduced in the medium‐ and high‐dose GH groups (*p* < 0.05). Within the GH groups, a dose‐dependent trend was observed, characterized by an increase in developing follicles and a decrease in atretic follicles; however, a statistically significant inter‐group difference was only identified for primary follicles (*p* < 0.05) (Table 3).

**TABLE 3:** Comparison of ovarian follicle counts of mice in each group.

Groups	Primary follicles	Secondary follicles	Antral follicles	Atretic follicles
Low‐dose GH group	36.00 ± 9.08^#,^*^,△^	52.60 ± 11.52*	24.60 ± 4.86^△^	47.90 ± 20.96
Medium‐dose GH group	43.30 ± 11.32^#,^*	52.20 ± 5.41*	32.70 ± 10.21	43.00 ± 11.26*^,△^
High‐dose GH group	54.30 ± 11.03*	61.50 ± 12.21*^,△^	34.30 ± 15.73	31.50 ± 5.99*
DOR group	25.40 ± 6.75^△^	38.70 ± 13.33^△^	23.00 ± 9.02^△^	69.50 ± 12.22^△^
NC group	49.20 ± 7.42	51.10 ± 10.76	34.30 ± 4.35	26.40 ± 6.06

*Note:* Continuous variables are presented as mean ± SD. *p* < 0.05 was considered as statistically significant.

^#^ *p* < 0.05, compared to high‐dose GH group. *p < 0.05, compared to DOR group. ^△^ *p* < 0.05, compared to NC group.

### 3.5 GH Treatment Modulated the Ovarian Transcriptomic Profile and Molecular Pathway

**FIGURE 3:**
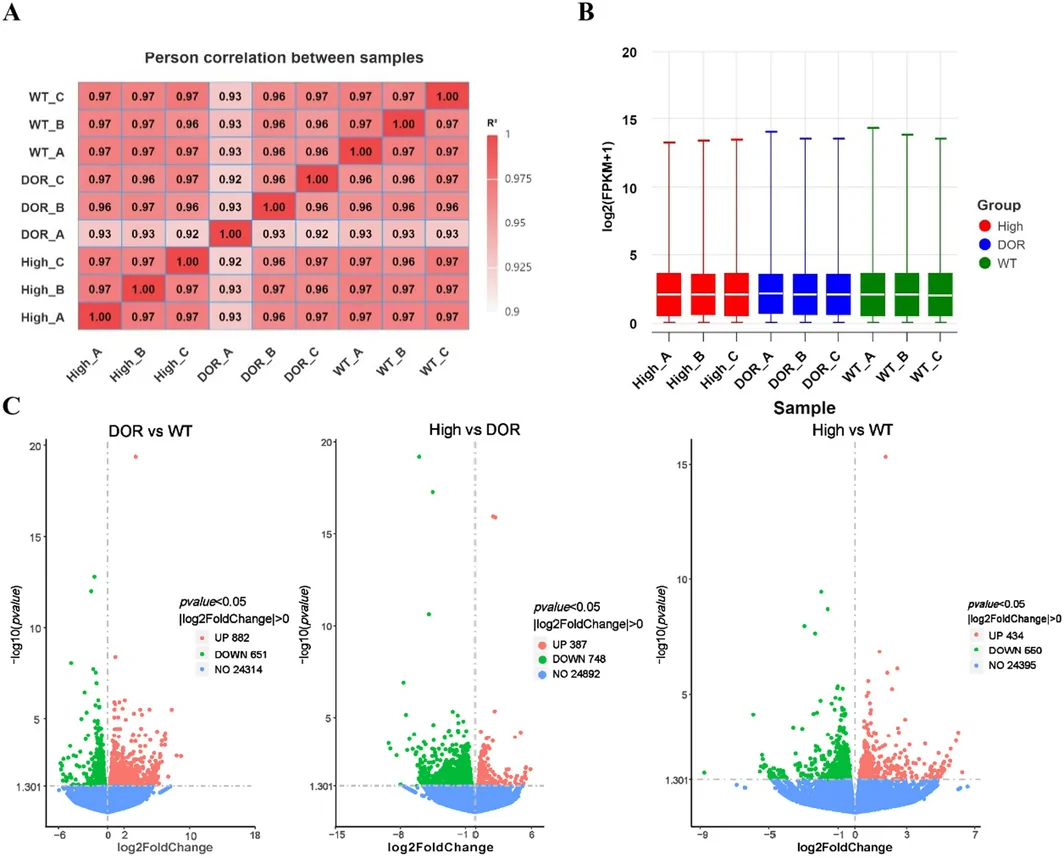
Analysis of differential gene expression between groups. High represents the high‐dose GH group; DOR represents the DOR group; *Wt* represents the NC group. (A) Both axes denote sample names, with numerical values in the table indicating correlation coefficients. A correlation coefficient approaching one indicates greater similarity in expression between samples. For the experiment, *R* ^2^ should be greater than 0.9. (B) Box plot of gene expression levels across samples, where the horizontal axis represents sample name and the vertical axis represents FPKM values. (C) Comparison of the number of DEGs among the different groups. The horizontal axis represents the fold change in gene expression between two groups, and the vertical axis represents the significance level of expression differences, with smaller adjusted *p* values indicating greater significance. Red dots represent upregulated genes, green dots indicate downregulated genes, and dashed lines denote the threshold for differential gene expression, with blue dots below this threshold indicating genes with no significant differential expression.

To explore the molecular mechanisms underlying the therapeutic effects of GH, we performed RNA‐Seq on the NC (Wt_A/B/C), DOR (DOR_A/B/C), and high‐dose GH (High_A/B/C) groups, the latter of which showed the most pronounced improvement in ovarian reserve. Pearson correlation coefficients (*R* ^2^) for all samples exceeded 0.9, confirming the high reproducibility and reliability of data for differential expression analysis (Figure 3A).

Gene expression levels were quantified as Fragments Per Kilobase of transcript per Million mapped fragments (Figure 3B). Comparative analysis identified 1533 differentially expressed genes (DEGs) between the DOR and NC groups (882 up‐regulated, 651 down‐regulated) (*p* < 0.05). Furthermore, 1135 DEGs were identified between the high‐dose GH and DOR groups (387 up‐regulated, 748 down‐regulated), while 984 DEGs were found between the high‐dose GH and NC groups (434 up‐regulated, 550 down‐regulated) (*p* < 0.05) (Figure 3C).

**FIGURE 4:**
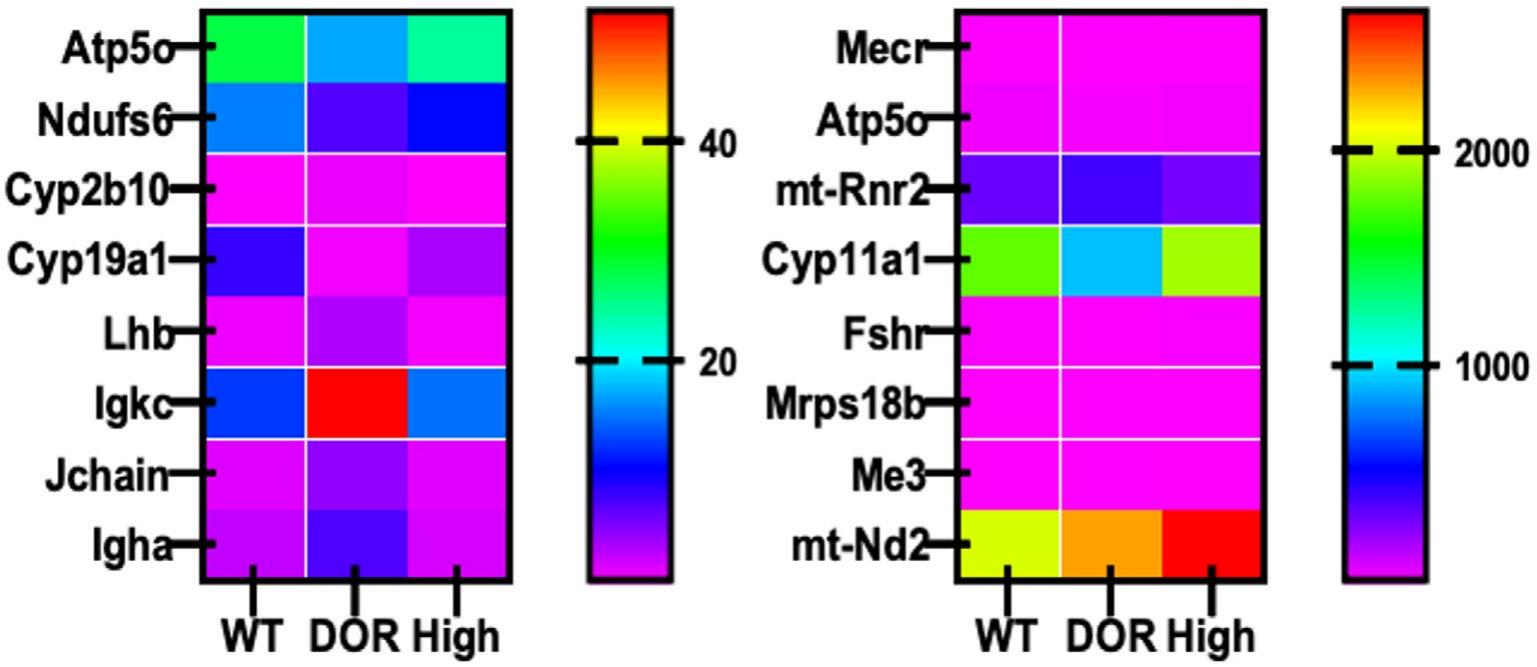
Heatmap demonstrated the restorative effect of GH on gene expression in ovarian tissue of DOR mice.

Functional analysis revealed that DEGs in the DOR group were primarily associated with mitochondrial metabolism (*Atp5o*, *Ndufs6*) and reproductive endocrine function (*Cyp2b10*, *Cyp19a1*, *LHB*) compared to the NC group. In contrast, DEGs in the high‐dose GH group compared to the DOR group were mainly involved in immune response (*Igkc*, *Jchain*, and *Igha*), mitochondrial metabolism (*Mecr*, *Atp5o*, mt‐Rnr2, and *Cyp11a1*), and reproductive endocrine function (*FSHR*). Compared to mice in the NC group, the DEGs in the high‐dose GH group were related mainly to mitochondrial metabolism (*Mrps18b*, *Me3*, *mt*‐*Nd2*) (Figure 4).

**FIGURE 5:**
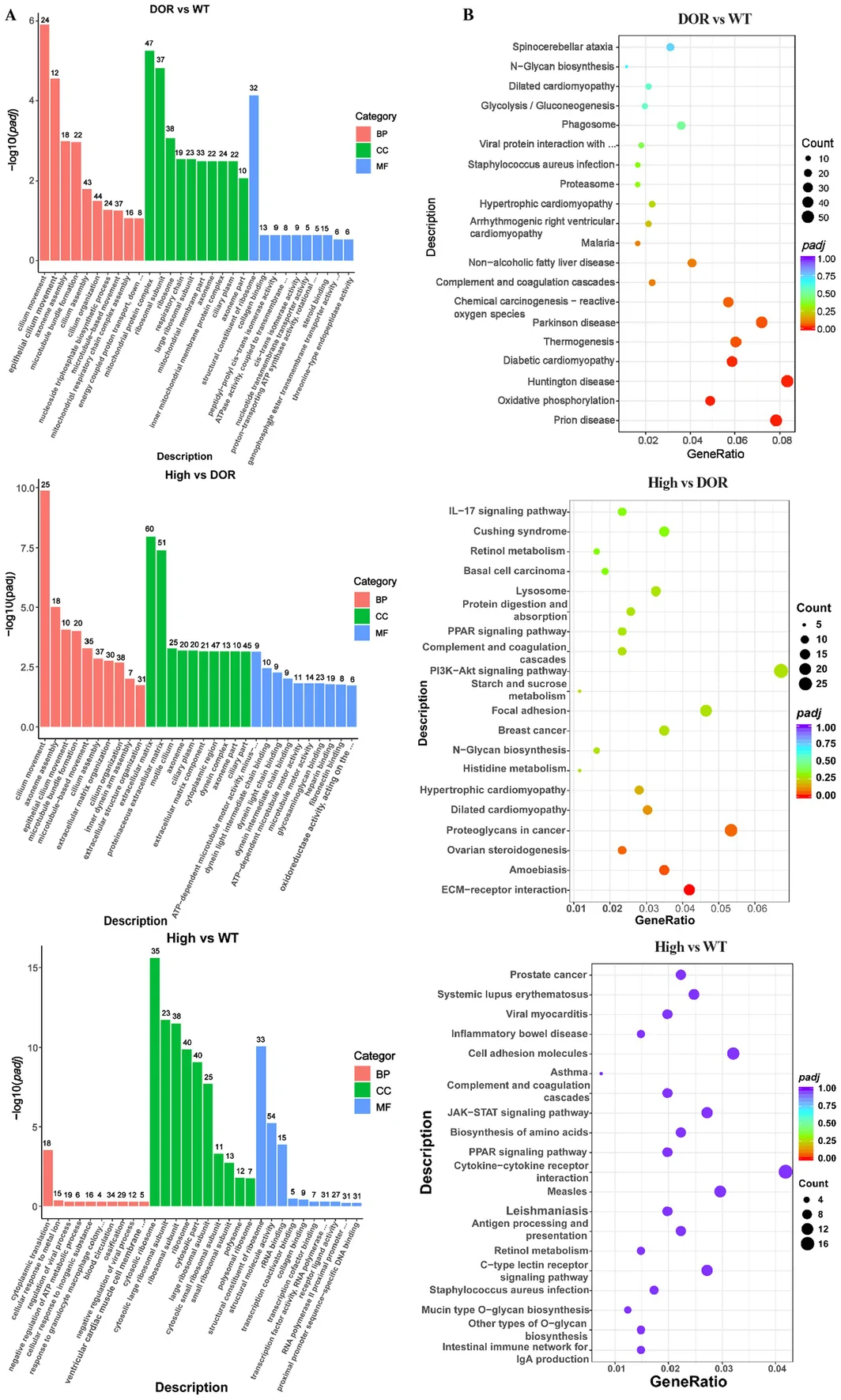
GO enrichment and KEGG signaling pathway analysis of the biological processes and signaling pathways by which GH improved ovarian function in DOR mice. High represents the high‐dose GH group; DOR represents the DOR group; *Wt* represents the NC group. Through the *t*‐test analysis, significantly differentially expressed genes between pairs of groups were identified. (A) In the GO enrichment analysis plot, the horizontal axis represents significantly enriched GO terms, and the vertical axis indicates the significance level of differential expression. The three GO subcategories are represented by different colors: red for BP (biological process), green for CC (cellular component), and blue for MF (molecular function), with higher numerical values indicating greater significance. (B) The horizontal axis represents the enrichment factor, and the vertical axis represents the KEGG pathways. The size of the dots indicates the number of genes annotated to each pathway, and the color ranging from red to purple represents the significance of the enrichment.

GO enrichment analysis revealed that the DOR group exhibited significant changes in ribosome and protein synthesis, mitochondrial metabolism and redox reactions compared to the NC group (*p* < 0.05). Following GH intervention, functions related to protein synthesis and oxidoreductase activity were significantly enriched (*p* < 0.05). Compared to the NC group, the high‐dose GH group exhibited significant enrichment related to ribosome synthesis (Figure 5A).

Notably, KEGG pathway analysis revealed that GH treatment significantly enriched pathways involving extracellular matrix interactions and steroid hormone synthesis compared to the DOR group (*p* < 0.05). No significantly enriched pathway was observed between the high‐dose GH and NC groups, suggesting a restoration of the transcriptomic profile (Figure 5B).

**FIGURE 6:**
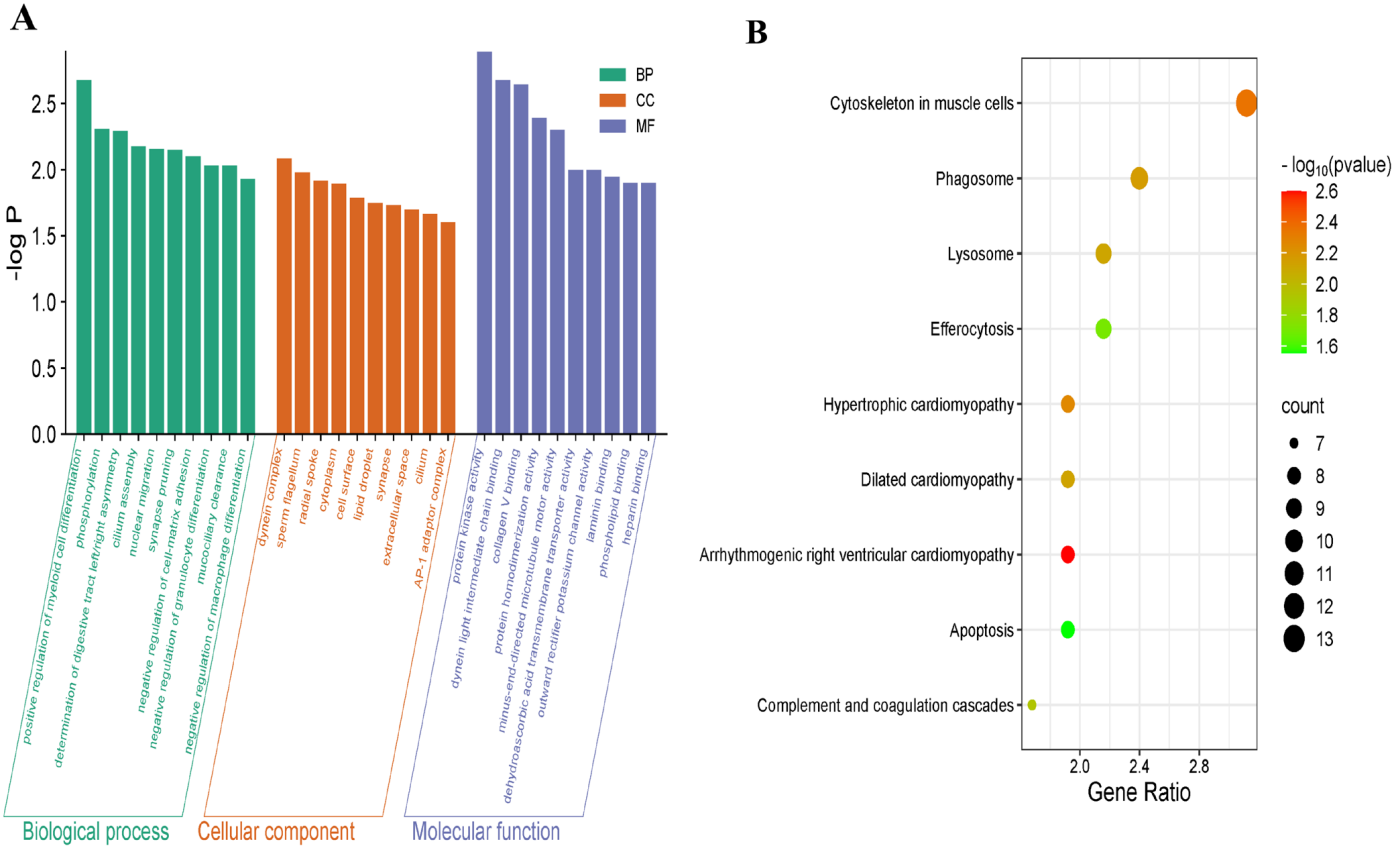
Reverse expression differential gene GO functional enrichment analysis and KEGG pathway enrichment analysis. (A) In the GO enrichment analysis plot, the horizontal axis represents significantly enriched GO terms, and the vertical axis indicates the significance level of differential expression. The three GO subcategories are represented by different colors: red for BP, green for CC, and blue for MF, with higher numerical values indicating greater significance. (B) The horizontal axis represents the enrichment factor, and the vertical axis represents the KEGG pathways. The size of the dots indicates the number of genes annotated to each pathway, and the color ranging from green to red represents the significance of enrichment.

A subset of key DEGs (e.g., *MAP2K2*, *FOS*, *BAK1*, *AK1*, *C1QA*, *EGR3*, *EPHA4*, *CDKL4*, *MRC2*, *CTSL*, *DDIT3*, *LMNA*, and *ATP6V0B*) demonstrated altered expression in the DOR group that was subsequently modulated by GH treatment. These genes were enriched across 30 GO terms and 9 KEGG pathways (Figure 6A,B). GO enrichment analysis categorized these genes into Biological Process (BP: phosphorylation, synapse pruning), cellular component (CC: dynein complex, synapse), and molecular function (MF: protein kinase activity). BP was enriched in processes such as phosphorylation, synapse pruning, and negative regulation of macrophage differentiation. CC was enriched in structures like dynein complex and synapse. MF has included functions such as protein kinase activity and outward rectifier potassium channel activity (Figure 6A). The enriched KEGG pathways primarily included phagosome, lysosome, apoptosis, complement, and coagulation cascades (Figure 6B).

## 4 Discussion

DOR has remained a formidable challenge for assisted reproduction. Patients with DOR typically yield fewer oocytes and embryos during IVF‐ET, ultimately leading to a suboptimal clinical pregnancy rate. While GH supplementation has shown efficacy in improving IVF‐ET outcomes, its precise molecular mechanisms in enhancing ovarian reserve have remained poorly characterized [29, 30, 31].

CDDP can establish DOR mice model within a shorter duration compared with CTX (7 days vs. 14 days) [27, 32]. CTX can suppress DNA synthesis, induce apoptosis, and senescence of granulosa cells, and consequently lead to follicular atresia [32]. CDDP directly triggers follicular damage and oocyte apoptosis [27]. Therefore, a CDDP‐induced DOR mice model was employed in our study for efficiency and direct effect. The folliculogenesis in mice (from primordial follicle activation to ovulation) was approximately 21 days [21, 28]. Therefore, a 21‐day GH intervention can encompass the full developmental cascade of a follicle cohort, providing an adequate therapeutic time window to rescue the chemotherapy‐induced ovarian depletion.

Our results demonstrated that GH administration could significantly increase the counts of primary and secondary follicles, with the most pronounced effects observed with the high dose (1.6 mg/kg/day). This aligns with clinical evidence indicating that GH co‐treatment during IVF cycles promotes antral follicle recruitment and suppresses apoptosis of granulosa cells [10, 25]. In contrast, Liu et al. [21] reported that a moderate‐dose GH (0.8 mg/kg) was sufficient to improve follicular development in CTX‐induced POF mice, whereas high‐dose GH (1.6 mg/kg) paradoxically increased atresia [21]. This discrepancy suggested that the therapeutic window for GH may shift depending on the cytotoxic agent used, necessitating tailored dose–response investigations across diverse ovarian injury phenotypes.

The ovary maintains systemic homeostasis through the secretion of steroid hormone. FSH, a pituitary‐derived gonadotropin, is essential for follicular maturation [33, 34]. Conversely, AMH, secreted by granulosa cells of preantral and small antral follicles, serves as a key gatekeeper of ovarian reserve by modulating FSH sensitivity and preventing follicular premature depletion [17, 35, 36]. Unlike the cyclic fluctuations of E_2_ and FSH, AMH levels remain stable throughout the estrous cycle [17, 37]. Clinically, DOR is characterized by reduced serum AMH and E_2_ levels alongside elevated FSH [2, 38]. Our findings align with previous reports demonstrating that 21‐day GH administration significantly increased E_2_ and decreased FSH in CTX‐induced POF mice [21]. However, Hou et al. [17] observed that GH failed to elevate AMH level in aged mice (32–34 weeks). This indicated that while physiological ovarian aging may be less responsive to GH, chemotherapy‐induced acute ovarian damage remains partially reversible.

The pathophysiology of DOR is intimately linked to oxidative stress and mitochondrial dysfunction [22, 39]. Previously, in vitro studies demonstrated that CDDP inhibits proliferation and triggers apoptosis in human granulosa cells by perturbing the *Bax/Bcl‐2* ratio and downregulating protective factors, such as *Bcl‐2*, *SirT3*, and Sod2 [40]. These molecular changes are typically accompanied by elevated reactive oxygen species levels, collapsed mitochondrial membrane potential, and decreased mitochondrial DNA copy numbers. By integrating our RNA‐Seq analysis, we hypothesize that CDDP can induce DOR by perturbing generic networks essential for mitochondrial metabolism (*Atp5o* and *Ndufs6*), oxidative phosphorylation, and reproductive function (*Cyp2b10*, *Cyp19a1*, and *LHB*). Notably, the downregulation of LHB is critical, as LH signaling is indispensable for steroidogenesis and folliculogenesis [41].

The restorative effect of GH is likely to involve multi‐pathways regulation of mitochondrial health, protein synthesis, and redox homeostasis [22, 42]. GH has been shown to reduce reactive oxygen species and restore mitochondrial integrity and modulate the *Bax/Bcl‐2* ratio to favor cell survival [40]. Furthermore, GH may modulate key developmental pathways, including the upregulation of *Notch‐1* and suppression of *Fos*/*Jun* activity, thereby inhibiting oocyte apoptosis [5, 21, 42, 43]. Our RNA sequencing analysis further elucidated this landscape, revealing that high‐dose GH can modulate genetic networks involved in mitochondrial metabolism (*Atp5o*, *mt‐Rnr2*, and *Cyp*), redox homeostasis, and the translational machinery (ribosome synthesis). Given that FSHR mutations or signaling failures are hallmark drivers of DOR pathogenesis [29], GH‐induced FSHR modulation may represent a significant therapeutic mechanism for sensitizing follicles to endogenous gonadotropin.

GO and KEGG enrichment analyses further underscore the importance of intercellular communication and metabolic homeostasis in ovarian health. The loss of germline‐soma communication (such as transzonal projections) is a hallmark of ovarian decline, which can be partially mitigated by antioxidants like melatonin [44]. These findings align with bioinformatics studies in other systems, where DEGs associated with cell communication and metabolic pathways (e.g., PI3K‐Akt signaling, extracellular matrix interactions) were identified as key regulators of tissue function [25]. Our identification of DEGs associated with cell communication and synaptic‐like connectivity suggests that GH may similarly preserve the complex signaling microenvironment of the follicle.

While our RNA‐Seq analysis has provided a robust transcriptomic foundation, we acknowledge that the limited sample size and the lack of extensive protein‐level validation (e.g., Western blotting or immunohistochemistry) are limitations of the current study. Future research should employ multi‐omics integration incorporating proteomics and metabolomics to further clarify how GH modulates mitochondrial flux and follicular survival pathways. Additionally, validating these dose–response relationships in other models of ovarian insufficiency will be crucial for translating these findings into optimized clinical GH supplementation protocols for the DOR patients.

## 5 Conclusions

GH can dose‐dependently enhance ovarian reserve in the DOR mice. This effect appears to be mediated by the modulation of genetic networks involved in mitochondrial energy metabolism, oxidative stress responses, and protein/ribosome biosynthesis.

## Author Contributions

Tianhong Huang: investigation, writing – original draft. Niu Wang: visualization, writing – original draft. Dongsheng Xiong: methodology. Yanan Zhang: investigation. Jiajing Wei: investigation. Furui Chen: investigation. Yu Qiu: investigation. Fangyi Long: formal analysis. Qianwen Luo: investigation. Jesse Li‐Ling: formal analysis. Yan Gong: conceptualization, funding acquisition, writing – review and editing.

## Funding

This study was jointly sponsored by the Science and Technology Innovation Program of Sichuan Maternal and Child Health Care Association (22FXZD01), Scientific Research Program of Sichuan Medical Association (S22015), Scientific Research Program of Chengdu Municipal Health Commission (2022155), National Center for Women and Children's Health, China CDC “Maternal and Infants Nutrition and Health Research Programs” (2023FYH012), and Natural Science Foundation of Sichuan Province (2024NSFSC0695).

## Ethics Statement

This study has been approved by the Animal Ethics Committee of Chengdu Medical College (approval no. 2023008), and all experiments have conformed to the national laws, regulations, and guidelines for the use of laboratory animals.

## Conflicts of Interest

The authors declare no conflicts of interest.

## Data Availability

The data from the results of this study are available from the corresponding author on reasonable request.
